# Hybrid genotype I and II ASFV *D250R* deletions confer protection against parental and genotype II strains and elicit potent immune response

**DOI:** 10.1080/22221751.2026.2640697

**Published:** 2026-03-13

**Authors:** Xiaoying Jia, Nan Li, Xuefei Sun, Junnan Ke, Min Zheng, Fengjie Wang, Huixian Yue, Zhuo Hao, Yiqian Jiang, Qixuan Li, Teng Chen, Yu Qi, Ying Wang, Shoufeng Zhang, Shuchao Wang, Rongliang Hu, Faming Miao, Yanyan Zhang

**Affiliations:** a State Key Laboratory of Pathogen and Biosecurity, Key Laboratory of Prevention & Control for African swine Fever and Other Major Pig Diseases, Ministry of Agriculture and Rural Affairs, Changchun Veterinary Research lnstitute, Chinese Academy of Agricultural sciences, Changchun, China; b College of Veterinary Medicine, Jilin University, Changchun, China; c College of Veterinary Medicine, Jilin Agricultural University, Changchun, China; d College of Life Sciences, Ningxia University, Yinchuan, China

**Keywords:** ASFV, genotype II, genotype I and II, gene-deleted mutant, attenuated, cross-protection, vaccine candidate

## Abstract

African Swine Fever Virus (ASFV) continues to mutate uncontrollably, with outbreaks caused by genotype II strains inflicting substantial economic losses on the global swine industry. Currently, a more virulent recombinant strain, combining genotype I and II, is spreading across China, Russia and Vietnam, exacerbating the severity of the epidemic. The development of safe and effective ASFV vaccines is therefore urgent. We constructed two gene-deleted strains – SY18ΔD250R (genotype II) and JX23-02ΔD250R (genotype I/II recombinant) – by knocking out the D250R gene from virulent field strains. Both strains were significantly attenuated and induced robust humoral and cellular immune responses in vaccinated pigs, with no clinical signs or mortality observed post-vaccination. Pigs immunized with SY18ΔD250R showed 80% protection (4/5 survived) against homologous SY18 challenge. Pig #60, which succumbed to infection, exhibited persistently negative humoral responses (*p54* antibody) and the lowest cell-mediated immunity (*IFN-γ* response) within the group. The vaccinated pigs demonstrated cross-protection against an attenuated genotype II strain but failed to resist heterologous challenge with genotype I/II ASFV strain, even after booster immunization. In contrast, JX23-02ΔD250R-immunized pigs achieved 100% survival against both the parental I/II recombinant strain and virulent genotype II strains, with rapid viral clearance and low tissue viral loads. Notably, both SY18ΔD250R and JX23-02ΔD250R exhibited low or undetectable shedding, viremia, and viral loads-offering a significant safety advantage. Thus, JX23-02ΔD250R represents a promising vaccine candidate with potential for application against circulating ASFV strains.

## Introduction

African Swine Fever (ASF) is a highly contagious, hemorrhagic, and invariably fatal swine disease. African Swine Fever Virus (ASFV) is the sole large double-stranded DNA virus that infects domestic pigs, wild boars, and soft ticks. As of October 2025, ASF has been reported in over 40 countries across five global regions. The virus has had a devastating impact on the global swine industry, resulting in substantial economic losses and posing a severe threat to global food security.

ASFV is a large, enveloped virus with a regular icosahedral structure, representing the sole member of the *Asfivirus genus* and *Asfarviridae family* [1]. The ASFV genome is linear double-stranded DNA, ranging from 170 to 194 kb in length, and encodes 150–195 open reading frames (ORF) involved in various functions such as viral replication, transcription, and immunosuppression [2]. Based on the B646L gene, 24 genotypes have been identified, with only genotype I and genotype II having widespread distribution outside of Africa [3]. The genome consists of variable terminal regions and a conserved central region. Significant variations in genome length among different genotypes occur due to large-scale insertions and deletions in the variable terminal regions, which also influence virulence [4–6]. The central conserved region exhibits greater than 98.5% identity [2].

Since ASFV's introduction to Georgia in 2007, followed by its spread throughout Europe and Asia, only genotype II has been detected. In August 2018, China reported its first outbreak of highly virulent genotype II ASFV [7]. In 2021, genotype I strains were reported [8], and in 2023, recombinant variants of ASFV genotype I and II were reported [9]. Animal experiments indicate that these strains exhibit increased virulence [9,10]. Subsequent reports have emerged from northern Vietnam and eastern Russia [11–13]. The high variability of ASFV genes, their potent transmissibility, and continuous evolution under strong selective pressure, coupled with human factors and the complexity of control efforts, collectively contribute to the frequent emergence of different genotypes and subtypes. Emergent variants significantly reduce the efficacy of existing vaccines, decrease diagnostic accuracy, and substantially increase the difficulty of epidemic prevention and control.

The development of African Swine Fever Virus (ASFV) vaccines is critical for addressing the threat of ASF. Research primarily focuses on inactivated vaccines, live vaccines (including naturally attenuated and artificially attenuated), and engineered vaccines. Currently, the most promising avenue is gene-deletion (artificially attenuated) live vaccines. Researchers have conducted numerous studies on ASFV gene deletions, such as the absence of genes like *MGFs*/*CD2v*, *I73R*, *I226R*, *I177L*, *A137R*, and *H240R*. Following these deletions, the virulence of the parental strain is completely reduced [14–19], and complete protection against homologous challenge can be achieved. Among all ASFV experimental vaccines tested to date, attenuated strains have demonstrated the best immunoprotective efficacy. Several live attenuated vaccines, based on genotype II strains, have achieved 100% immunoprotective efficiency. However, ASFV exhibits a remarkable rate of mutation, rendering genotype II-based attenuated live vaccines ineffective against current circulating genotype I/II strains [20].

*D250R* (g5R) functions as a virus-specific decapping enzyme that accelerates the degradation of host mRNA by removing its 5' cap structure, ultimately inducing host protein synthesis shutoff. This mechanism not only facilitates the viral hijacking of the host translational machinery but, more critically, enables the virus to evade innate immune defenses by blocking the synthesis of antiviral proteins, such as interferons. Consequently, the deletion of *D250R* abrogates the viral suppression of host antiviral responses, thereby resulting in viral attenuation while simultaneously enhancing immunogenicity [21–24]. This study generated the SY18ΔD250R and JX23-02ΔD250R strains, characterized by the deletion of the *D250R* gene. Both strains exhibited completely reduced virulence, induced high levels of humoral and cellular immunity, and conferred protection against parental strain challenge. The JX23-02ΔD250R strain demonstrated cross-protection and represents a promising vaccine candidate for combating circulating genotype II and genotype I and II ASFV.

## Results

### *D250R* gene homology analysis

**Figure 1.**
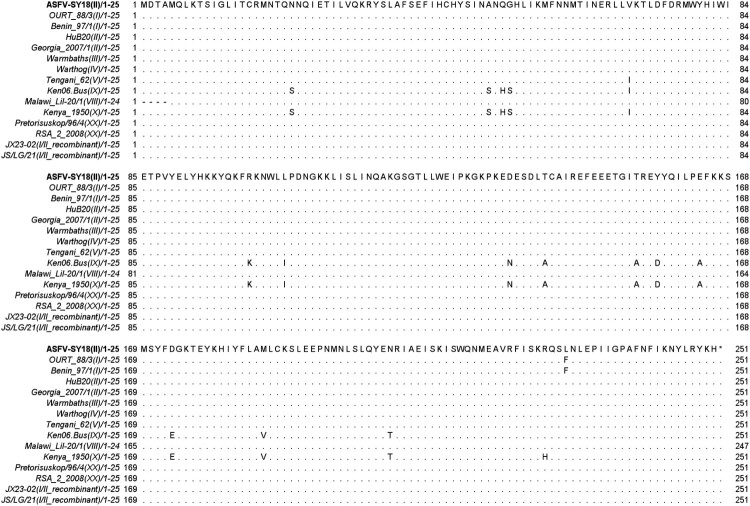
Homology comparison of *D250R* amino acids across different ASFV genotypes, indicates complete amino acid identity among the compared ASFV strains. – , denotes the absence of an amino acid in a particular strain compared to other genotype strains. Amino acids that are independently displayed, such as I and S, denote positions of amino acid divergence in this strain when compared to other genomes.

Using the ASFV SY18 strain, isolated from the first ASFV outbreak in China in 2018, as the reference sequence, amino acid residues of the *D250R* gene from 15 ASFV strains representing 10 genotypes were compared for homology (Figure 1). The results indicated that the homology of *D250R* amino acid residues among different genotypes ranged from 93.2% to 100%. The shortest *D250R* was found in Malawi Lil-20/1 (VIII), encoding 247 amino acids. Apart from a deletion of 4 amino acids at the N-terminus, the remaining amino acids showed no frameshift or mutations. The lowest homology was observed in the Ken06.Bus (IX) and Kenya 1950 (X) strains, with 93.6% homology to the reference genome. This was followed by OURT 88/3 (I) and Benin 97/1 (I), which showed 99.6% *D250R* amino acid homology. In contrast, the *D250R* amino acid homology was 100% for the HuB20 (II), ASFV Georgia 2007/1 (II), Warmbaths (III), Warthog (IV), Tengani 62 (V), Malawi Lil-20/1 (VIII), Pretorisuskop/96/4 (XX), RSA_2_2008 (XX), JX23-02 (I/II), and JS/LG/21 (I/II) strains. The comparative analysis demonstrated that *D250R* is highly conserved among different genotypes of ASFV.

### Reconstitution of *D250R* gene deletion strains

**Figure 2.**
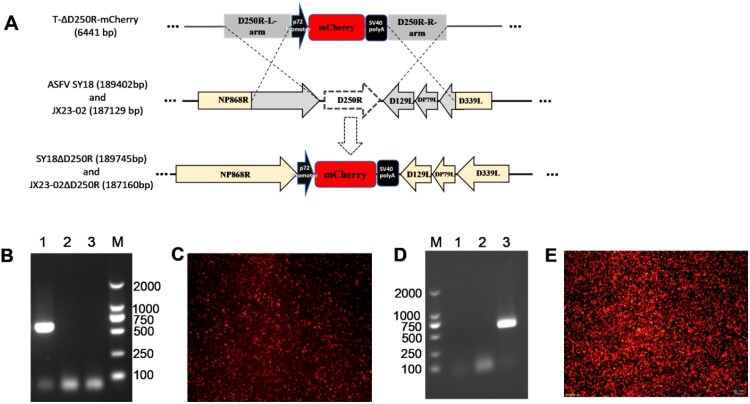
Generation and identification of *D250R* deletion virus strains. A: Schematic diagram illustrating the recombination process of the engineered virus within cells. B: Purification and identification of SY18ΔD250R. Lane 1 shows PCR amplification of the *D250R* gene from ASFV SY18, yielding a 630 bp target fragment. Lane 2 is a blank control (negative). Lane 3 shows PCR amplification of the *D250R* gene from SY18ΔD250R, with no target fragment detected. M represents the DL 2000 Marker. C: Fluorescence images of BMDMs infected with SY18ΔD250R at a MOI of 0.5 for 72 h. D: Purification and identification of JX23-02ΔD250R. M represents the DL 2000 Marker. Lane 1 is a blank control. Lane 2 shows the *D250R* gene from JX23-02ΔD250R. Lane 3 shows the *D250R* gene from JX23-02. E: Fluorescence images of BMDMs infected with JX23-02ΔD250R at a MOI of 0.5 for 72 h.

Donor plasmids underwent homologous recombination with the ASFV SY18 and ASFV JX23-02 genomes in cells, resulting in the replacement of the *D250R* gene with a *p72* promoter-mCherry-SV40 polyA sequence (Figure 2(A)). This yielded mCherry-tagged deletion viruses SY18ΔD250R and JX23-02ΔD250R. Following multiple rounds of serial endpoint dilution and observation under fluorescence microscopy, no *D250R* gene fragments were detected in the viral supernatant of SY18ΔD250R and JX23-02ΔD250R deletion strains (Figure 2(B and D)), indicating the absence of parental strain contamination in both gene deletion viruses. Purified SY18ΔD250R and JX23-02ΔD250R strains were used to infect BMDMs at a multiplicity of infection (MOI) of 0.5. Infected cells from both strains expressed red fluorescence. At 72 hours post-infection (hpi), the infection rate of cells inoculated with JX23-02ΔD250R exceeded 90%, while the infection rate of cells inoculated with SY18ΔD250R was less than 60%. This demonstrates that JX23-02ΔD250R exhibits a significantly higher infection efficiency than SY18ΔD250R (Figure 2(C and E)).

### Growth characteristics and genome changes of gene deletion viruses

**Figure 3.**
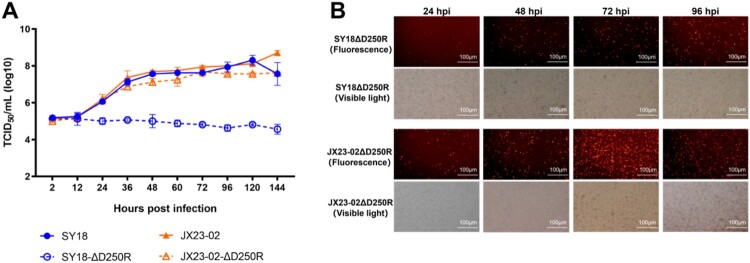
Growth Characteristics and Genome Sequencing Analysis of Gene Deletion Viruses. A: In vitro growth curves of gene deletion viruses and wild-type strains. B: Fluorescence imaging results of deletion viruses.

SY18ΔD250R, JX23-02ΔD250R, and their parental strains ASFV SY18 and JX23-02 were used to infect PAMs at a MOI of 0.01. Viral titres were collected and measured at different time points, and growth curves were plotted (Figure 3(A)) to compare the in vitro growth characteristics of the *D250R* gene deletion viruses with their parental strains. The results showed no significant difference in replication between JX23-02ΔD250R and its parental strain JX23-02 in PAMs at all time points. However, a significant difference was observed between SY18ΔD250R and its parental strain ASFV SY18 starting from 24 hpi (*p* < 0.05). Throughout the infection of BMDM cells with SY18ΔD250R at a MOI of 0.01, the viral titre did not significantly increase or decrease, indicating that the absence of the *D250R* gene significantly affected the replication capacity of ASFV SY18. A comparison of fluorescence images from PAMs infected with SY18ΔD250R and JX23-02ΔD250R also revealed that the proportion of fluorescent cells infected by SY18ΔD250R was lower than that infected by JX23-02ΔD250R, suggesting that the deletion of the *D250R* gene had differential impacts on the replication capacities of ASFV SY18 and JX23-02 strains (Figure 3(B)).

Next-generation sequencing revealed that the genome sequence of SY18ΔD250R was identical to its parental strain ASFV SY18, with the exception of the deleted *D250R* gene. The open reading frame (ORF) sequences of the JX23-02ΔD250R genome were identical to those of the JX23-02 strain. However, 10 nucleotide mutations were identified within the non-coding regions. These mutations did not result in the creation of new ORFs. Detailed information on the mutations is provided in Table 1.

**Table 1.** Summary of genomic mutations identified in the JX23-02ΔD250R mutant compared to the parental strain.

Genomic Loci	JX23-02 (Reference genome)	JX23-02ΔD250R (Modified genome)	Mutational region
45	A	T	Left Variable Region
566	A	–	Left Variable Region
989	T	C	Left Variable Region
16851	G	T	Central Conservative Region
16884	T	G	Central Conservative Region
186754	A	G	Right Variable Region
186755	C	T	Right Variable Region
186756	C	T	Right Variable Region
186759	T	A	Right Variable Region
186764	C	T	Right Variable Region

Note: –, indicates a deletion of the gene at this position.

### Clinical signs, body temperature, and survival rate in experiment 1

**Figure 4.**
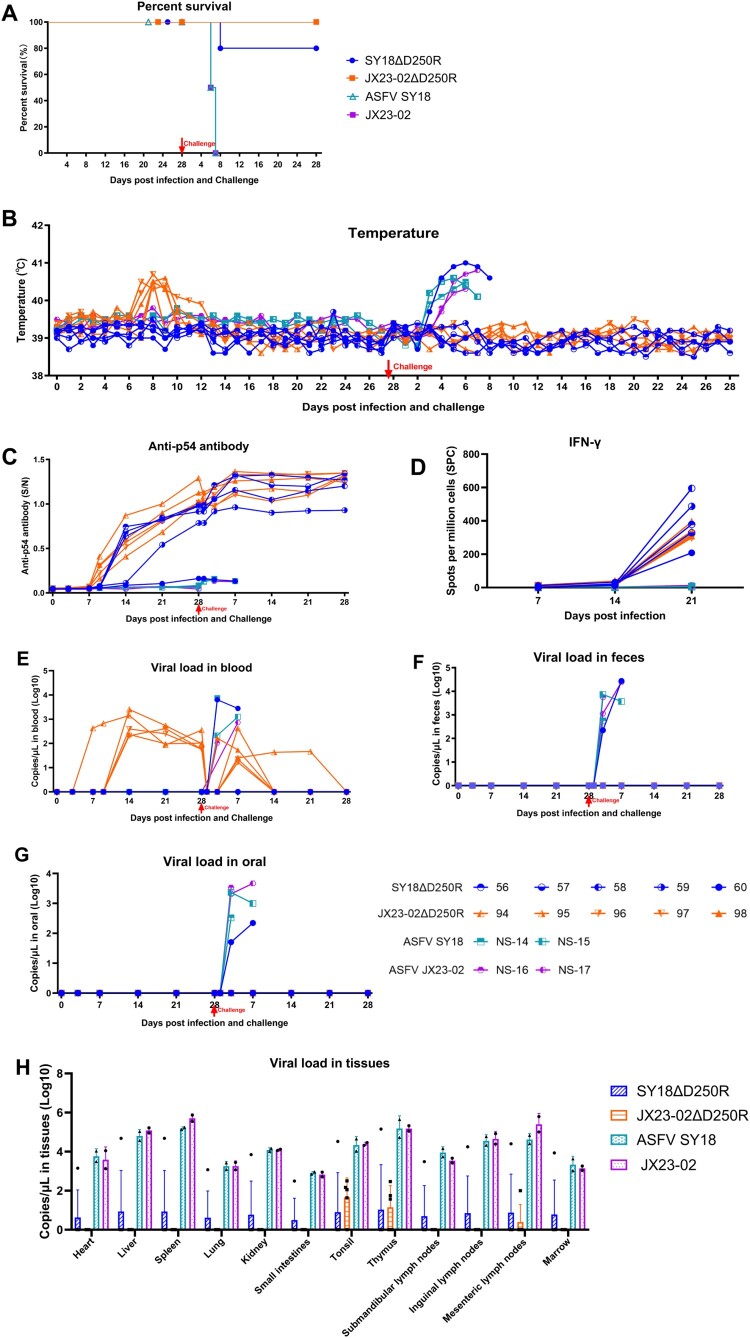
Experiment 1: Immunization-Challenge Protection Experiment. A: Survival rate of pigs during immunization and challenge. B: Rectal temperature changes in pigs during immunization and challenge. C: *p54* antibody levels in pigs during immunization and challenge. D: Number of cells specifically secreting *IFN-γ* cytokine per million peripheral blood mononuclear cells during immunization. E, F, G: Copy number of ASFV nucleic acid detected in blood, saliva, and feces, respectively, during immunization and challenge. H: Viral load detection in all experimental pig tissues.

During the observation period, all five pigs in Group A (immunized with SY18D250R) maintained normal body temperature, appetite, and clinical presentation, with 100% survival. In Group B (immunized with JX23-02D250R), all five pigs exhibited sustained fever for three days (peaking at 40.7°C) between 7 and 9 days post-immunization (dpi), after which their body temperatures returned to normal, resulting in 100% survival. During the period of elevated temperature, there was a slight decrease in appetite, which recovered to normal along with their mental state once their body temperature normalized. Pigs in Group C (control) showed normal body temperature, appetite, and clinical signs, with 100% survival (Table 2, Figure 4(A and B)).

**Table 2.** Fever and survival rate during immunization period.

Groups	Immunity	Fever: Mean			No. of survivors	Mean days to death [±SD]
		Onset (Day) [±SD]	Duration (Day)[±SD]	Highest rectal temp (°C)		
A	10^5.0^TCID_50_ SY18ΔD250R	/	/	/	5/5	/
B	10^5.0^TCID_50_ JX23-02ΔD250R	7.6 [±0.548]	2.4[±1.14]	40.7	5/5	/
C	0.9% NaCl	/	/	/	4/4	/

Note: “/” indicates absence of this condition, [SD] stands for Standard Deviation.

During the parental strain challenge period, one pig (No. 60) in Group A developed fever at 4 days post-challenge (dpc) and exhibited severe classical African Swine Fever Virus (ASFV) symptoms at 8 dpc, leading to euthanasia. The other four pigs maintained normal body temperature, appetite, and clinical presentation, with an 80% survival rate. All five pigs in Group B presented with normal body temperature, appetite, and clinical signs, achieving 100% survival. In contrast, pigs in Group C, after being challenged with the SY18 and JX23-02 strains, developed severe classical ASFV symptoms at 6 dpc and 7 dpc, respectively, and were euthanized, resulting in zero survivors. This confirms the validity of the challenge control results (Table 3, Figure 4(A and B)).

**Table 3.** Fever and survival rate during challenge period.

Groups	Challenge	Fever			Survivors/Total	Death days (Day) [±SD]
		Onset (Day) [±SD]	Duration (Day) [±SD]	Highest rectal temp (°C)		
A	10^2.0^TCID_50_ SY18	4.0[±0]	4.0[±0]	41.0	4/5	4.0[±0]
B	10^2.0^TCID_50_ JX23-02	/	/	/	5/5	/
C	10^2.0^TCID_50_ SY18	3.5[±0.707]	4.0[±1.414]	40.6	0/2	6.5[±0.707]
	10^2.0^TCID_50_ JX23-02	5.0[±0]	2.5[±0.707]	40.7	0/2	6.5[±0.707]

Note: /, indicates absence of this condition, [SD] stands for Standard Deviation.

### Viral load detection in experiment 1

During the immunization observation period, ASFV nucleic acid was not detected in the saliva, feces, or blood of the five pigs in Group A. In Group B, ASFV nucleic acid was not detected in saliva or feces, but it was detected in the blood of all five pigs. Further identification confirmed that the nucleic acid detected in these blood samples belonged to the vaccine strain (Supplementary File 2). Pigs in Group C did not receive viral immunization and no viral nucleic acid was detected (Figure 4(E, F, and G)).

During the challenge observation period, with the exception of pig No. 60 which was euthanized due to the challenge, ASFV nucleic acid was not detected in the saliva, feces, or blood of the four surviving pigs in Group A. In Group B, ASFV nucleic acid was not detected in oral or anal swabs from all five pigs, but it was detected in their blood. The dentification confirmed that the nucleic acid detected in these blood samples belonged to the mixture of the vaccine strain and the challenge strain (Supplementary File 2). However, after 14 dpc, ASFV nucleic acid was undetectable in the blood of 4 out of 5 pigs (Figure 4(E, F, and G)).

Twenty-eight days post-challenge, at the end of the observation period, all surviving pigs were euthanized. Tissues such as the heart, liver, spleen, and lymph nodes were collected and analyzed by qPCR. Compared to the tissue viral load results in Group C (control), pigs in Group A showed lower overall viral loads in their tissues after immunization and challenge. In Group B, only low levels of viremia were detected in the tonsils, thymus, and mesenteric lymph nodes (Figure 4(H)). The viral nucleic acid in the tissues is a mixture of the vaccine strain and the challenge strain (Supplementary File 2).

### Humoral and cellular immune response detection in experiment 1

Humoral immunity was assessed by detecting *p54* antibodies using ELISA. The results indicated that pigs in Groups A and B seroconverted (S/N > 0.25) starting at 9 dpi, with antibody levels reaching their peak at 28 dpi (Figure 4(C)). Following challenge, antibody levels remained at their highest. Cellular immunity was evaluated by detecting the number of peripheral blood mononuclear cells secreting *IFN-γ* via ELISpot assay at 7 dpi, 14 dpi, and 21 dpi. The results showed that by 21 dpi, pigs in both Groups A and B developed cellular immunity against ASFV (Figure 4(D)).

Notably, in the SY18ΔD250R-immunized group, the sole non-survivor (#60) remained consistently negative for *p54*-specific antibodies. Although its cellular immune response IFN-γSPC>208 was positive, it was the lowest among all immunized pigs. This suggests that humoral immunity is closely linked to protection and acts synergistically with cellular immunity to provide dual-layer immune defense.

### Necropsy and pathological analysis in experiment 1

**Figure 5.**
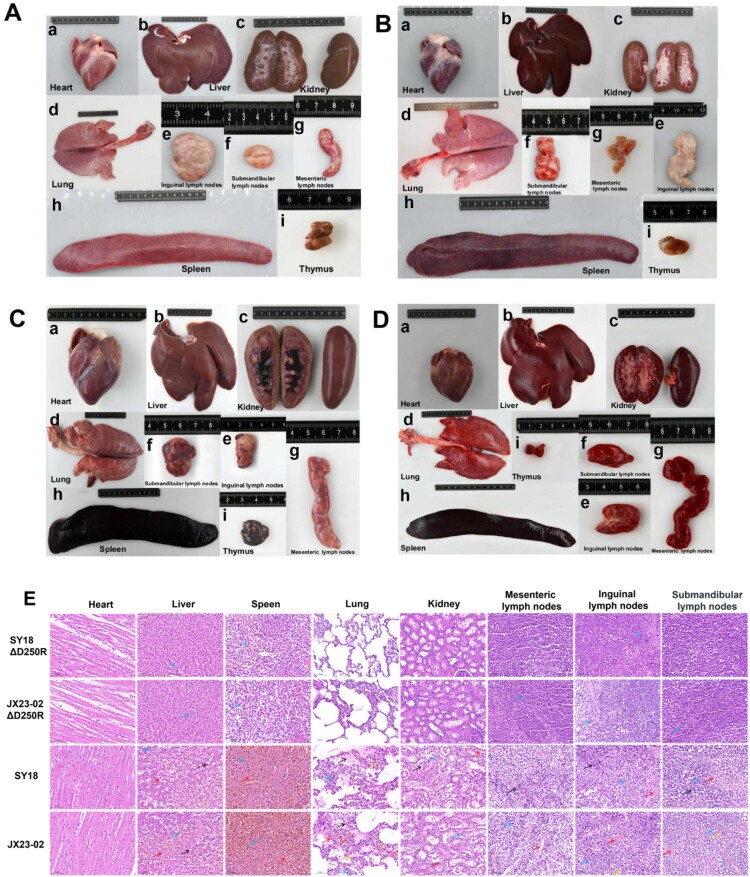
Gross and Pathological Analysis of Tissues. A: Gross pathology of surviving pigs immunized and challenged with SY18ΔD250R. B: Gross pathology of surviving pigs immunized and challenged with JX23-02ΔD250R. C: Gross pathology of control pigs challenged with SY18. D: Gross pathology of control pigs challenged with JX23-02. E: Histopathological changes in tissues from immunized and control pigs (H&E staining). Representative images show severe lesions in the challenge control groups (#14, #17) compared to the normal histology of the vaccine groups (#58, #95). Red arrows indicate severe congestion and diffuse hemorrhage (observed in liver, spleen, lung, kidney, and lymph nodes). Blue arrows indicate infiltration of inflammatory cells. Black arrows indicate specific lesions depending on the tissue: fatty degeneration in the liver; fibrinous exudates in the lung; necrosis of epithelial cells in the kidney; and lymphocyte depletion or hyperplasia in lymph nodes. Yellow arrows indicate thickening of the pulmonary interstitium or hyperplasia in lymph nodes.

Post-challenge, pigs in Group A (immunized with SY18ΔD250R) and Group B (immunized with JX23-02ΔD250R) exhibited no characteristic hemorrhagic lesions associated with ASFV in any parenchymal organs, and the gross morphology and structure of the organs remained essentially normal. In sharp contrast, pigs in Group C (challenged with SY18) and Group D (challenged with JX23-02) displayed severe systemic hemorrhage, congestion, and tissue necrosis across all organs, consistent with the pathological characteristics of typical acute African Swine Fever. Specifically, the spleen exhibited extremely severe hemorrhagic splenomegaly (Figure 5(Ch and Dh)). Lymph nodes presented with severe hemorrhagic lymphadenitis (Figure 5(Cefg and Defg)). The kidneys were swollen and darkened (Figure 5(Cc and Dc)); the cut surface revealed severe congestion and hemorrhage, characterized by scattered or diffuse petechial hemorrhages in the cortex and deep dark-red congestion in the medulla, which obscured the structural architecture. The lungs showed signs of interstitial pneumonia and severe pulmonary edema (Figure 5(Cd and Dd)). The liver appeared congested and enlarged, presenting a dark reddish-brown colour with blunt edges (Figure 5(Cb and Db)), and the thymus displayed distinct scattered or diffuse petechial hemorrhages (Figure 5(Ci and Di)).

Histopathological examination (H&E staining) further confirmed the gross pathology results (Figure 5(E)). In the challenge control groups, severe tissue damage was observed across multiple organs. The spleen exhibited extensive hemorrhage, severe congestion of the red pulp, and almost complete depletion of the white pulp. The liver showed sinusoidal congestion, fatty degeneration, and inflammatory cell infiltration. In the lungs, we observed severe interstitial pneumonia characterized by thickened alveolar septa, destruction of alveolar architecture, and the accumulation of inflammatory cells and fibrinous exudates within the alveolar spaces. The kidneys displayed necrosis of renal tubular epithelial cells accompanied by hemorrhage. Lymph nodes, particularly the inguinal and submandibular nodes, presented with severe hemorrhage and lymphocyte depletion.

In stark contrast, pigs in Groups A (SY18ΔD250R) and B (JX23-02ΔD250R) that survived the challenge maintained normal tissue architecture. No significant hemorrhage, necrosis, or severe pathological lesions were observed in the heart, liver, spleen, lungs, kidneys, or lymph nodes. Mild infiltration of inflammatory cells was occasionally noted in the liver, spleen, and lymph nodes, indicative of an active immune response rather than viral-induced damage. However, Pig #60 in Group A, which succumbed to the challenge, exhibited severe systemic hemorrhagic and necrotic lesions indistinguishable from those in the control group (Data not shown).

### Clinical signs, body temperature, and survival rate in experiment 2

**Figure 6.**
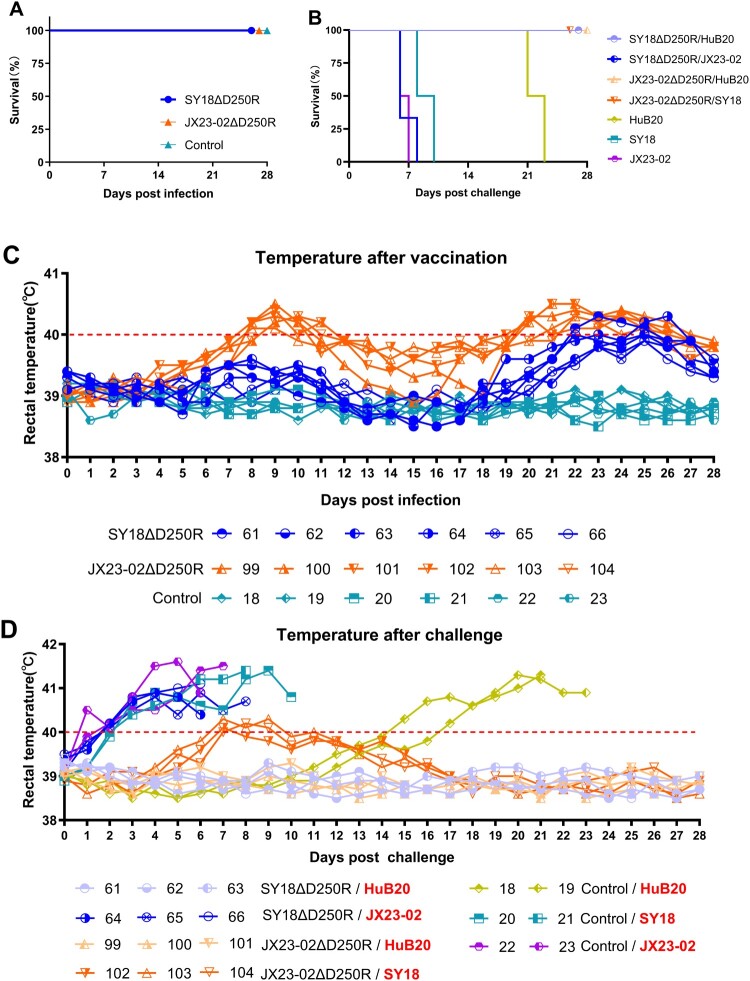
Cross-Challenge Protection Experiment. A: Survival rates of each group during immunization. B: Survival rates during cross-challenge. C: Body temperature changes during immunization. D: Body temperature changes during cross-challenge.

During the immunization observation period, in Group D (immunized with SY18ΔD250R), six pigs showed no fever or clinical abnormalities within 14 days post-primary immunization. On day 8 after booster immunization, 3 out of 6 pigs experienced low-grade fever (maximum 40.3°C) for 1–4 days, with normal clinical presentation and complete survival. In Group F (immunized with JX23-02ΔD250R), all six pigs showed a consecutive 3-day fever (maximum 40.5°C) on day 8 post-primary immunization. On day 5 post-booster immunization, all pigs experienced low-grade fever (maximum 40.5°C) for 5–7 days. Pigs that developed fever after booster immunization showed decreased appetite; their appetite and mental state returned to normal after body temperature normalized. Pigs in Group C (control group) maintained normal temperature, appetite, and clinical presentation (Table 4, Figure 6(A and C)).

**Table 4.** Fever and survival rate during immunization period.

Groups	Immunity	Primary immunization			Booster immunization			Survivors/Total	Death days [SD]
		Onset of Fever (Day) [SD]	Duration of Fever (Day) [SD]	Highest rectal temp (°C)	Onset of Fever (Day) [SD]	Duration of Fever (Day) [SD]	Highest rectal temp(°C)		
D	10^5.0^TCID_50_ SY18ΔD250R	/	/	39.6	23.5 [±2.12]	3.0 [±1.41]	40.3	6/6	/
E	10^5.0^TCID_50_ JX23-02ΔD250R	8.33 [±0.52]	2.83 [±0.75]	40.5	20.5 [±0.84]	5.83 [±1.17]	40.5	6/6	/
F	0.9% NaCl	/	/	/	/	/	/	6/6	/

Note: “/” indicates absence of this condition, [SD] stands for Standard Deviation.

During the cross-challenge observation period, pigs in Group F (challenge control group) succumbed to severe typical ASFV symptoms. Two pigs challenged intramuscularly with 10^5.0^ TCID_50_ HuB20 were euthanized on 21 and 23 days post-challenge (dpc). Two pigs challenged intramuscularly with 100 TCID_50_ SY18 were euthanized on 8 and 10 dpc. Two pigs challenged intramuscularly with 100 TCID_50_ JX23-02 were euthanized on 6 and 7 dpc. In Group D, pigs 61, 62, and 63 (immunized with SY18ΔD250R) challenged intramuscularly with 100 TCID_50_ HuB20 showed no increase in body temperature or abnormal clinical signs and all survived. Pigs 64, 65, and 66 (immunized with SY18ΔD250R) challenged intramuscularly with 100 TCID_50_ JX23-02 exhibited severe typical ASFV symptoms on 6 and 9 dpc and were euthanized. In Group E, pigs 99, 100, and 101 (immunized with JX23-02ΔD250R) challenged intramuscularly with 10^5.0^ TCID_50_ HuB20 showed no increase in body temperature or abnormal clinical signs and all survived. Pigs 101, 102, and 103 (immunized with JX23-02ΔD250R) challenged intramuscularly with 100 TCID_50_ SY18 experienced low-grade fever (maximum 40.3°C) for 1–3 days on 7 dpc, followed by a return to normal body temperature. They displayed no abnormal clinical signs and all survived (Table 5, Figure 6(B and D)).

**Table 5.** Fever and survival rate during challenge period.

Groups	Immunity	Challenge	Fever			No. of survivors	Mean days to death [SD]
			Onset (Day) [SD]	Duration (Day)[SD]	Highest rectal temp (°C)		
D	10^5.0^TCID_50_ SY18ΔD250R Prime-boost (D0-D14)	10^5.0^ TCID_50_ HuB20 (II), IM, (D28)	/	/	/	3/3	/
		100 TCID_50_ JX23-02 (I/II), IM, (D28)	2.0 [±0]	5.67 [±1.16]	40.8	0/3	6.67 [±1.16]
E	10^5.0^TCID_50_ JX23-02ΔD250R Prime-boost (D0-D14)	10^5.0^ TCID_50_ HuB20 (II), IM, (D28)	/	/	/	3/3	/
		100 TCID_50_ SY18 (II), IM, (D28)	7.33 [±0.58]	2.0 [±1.0]	40.3	3/3	/
F	0.9% NaCl (D0-D14)	10^5.0^ TCID_50_ HuB20 (II), IM, (D28)	16.0 [±1.41]	7.0 [±2.83]	41.3	0/2	22.0 [±1.41]
		100 TCID_50_ SY18 (II), IM, (D28)	3.0 [±0]	7.0 [±1.41]	41.4	0/2	9.0 [±1.41]
		100 TCID_50_ JX23-02 (I/II), IM, (D28)	1.5 [±0.71]	6.0 [0]	41.6	0/2	6.5 [±0.71]

Note: “/” indicates absence of this condition, [SD] stands for Standard Deviation.

### Antibody and viral load in experiment 2

**Figure 7.**
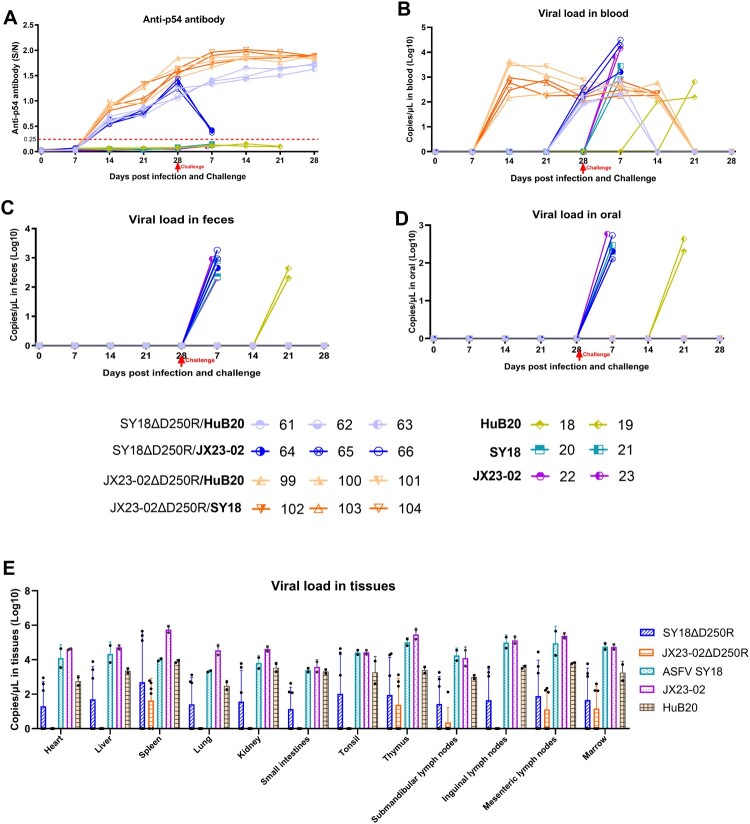
Antibody and Viral Load. A: *p54* antibody detection during immunization and challenge. B: Viremia detection during immunization and challenge. C: Fecal shedding detection during immunization and challenge observation period. D: Salivary shedding detection during immunization and challenge observation period. E: Viral load in tissues detection

The antibody trends observed during the immunization period in this experiment were similar to those in Experiment 1. However, pigs immunized with SY18ΔD250R and challenged with JX23-02 succumbed to the infection by day 8 dpc, with anti-*p54* antibody levels showing a rapid decline. In contrast, pigs challenged with SY18 all survived, and their anti-*p54* antibody continued to rise (Figure 7(A)).

Viremia during the immunization period mirrored Experiment 1, with JX23-02ΔD250R immunized pigs exhibiting persistently low levels of viremia. Notably, 50% of SY18ΔD250R immunized pigs also displayed low-level viremia during this phase. Following challenge, viral nucleic acid was undetectable in the blood of JX23-02ΔD250R immunized pigs after 21 dpc, and in SY18ΔD250R immunized pigs after 14 dpc (Figure 7(B)). During immunization, viral nucleic acid was not detected in feces or saliva of pigs immunized with either JX23-02ΔD250R or SY18ΔD250R. Post-challenge, surviving pigs also showed no detectable viral nucleic acid in their tissues or saliva (Figure 7(C and D)).

At the end of the observation period, viral loads in the tissues of affected and surviving pigs were analyzed. Compared to the control group, pigs immunized with SY18ΔD250R and protected from challenge exhibited lower viral loads in their tissues. However, pigs immunized with JX23-02ΔD250R and protected from challenge showed even lower viral burdens. In the tissues examined, low-level viral nucleic acid was detected only in the spleen, thymus, submandibular lymph nodes, mesenteric lymph nodes, and bone marrow of some pigs (Figure 7(E)). Further identification confirmed that the nucleic acid detected in these tissues belonged to the mixture of the vaccine strain and challenge strain (Supplementary File 2).

## Discussion

This study is the first to identify *D250R* as a critical African swine fever virus (ASFV) virulence gene, which is highly conserved across different ASFV genotypes. By deleting the *D250R* gene, we constructed two vaccine candidates based on the earliest genotype II strain prevalent in China (SY18) and a recently emerged genotype I/II recombinant strain (JX23-02). Comparing these strains in vitro, we observed distinct replication kinetics: while SY18ΔD250R replication was limited, JX23-02ΔD250R exhibited significantly superior replication rates. Further analysis revealed 10 nucleotide mutations in the non-coding regions of JX23-02ΔD250R, which were confirmed to be stable during plaque purification. While non-coding region variations are common in ASFV, whether these specific mutations influence viral replication kinetics or immune recognition warrants future verification through site-directed mutagenesis.

Although both SY18ΔD250R and JX23-02ΔD250R showed significant attenuation in vivo, their safety profiles presented notable differences. Specifically, pigs inoculated with JX23-02ΔD250R developed a transient low-grade fever following both primary and booster immunizations. This phenomenon suggests that the integration of Type I and Type II genomes confers a stronger innate replication advantage and baseline virulence to the chimeric recombinant strain compared to the pure Type II backbone. This backbone-dependent virulence disparity is further supported by our brief observation with another target: while a pure Type II strain lacking the I226R gene was highly attenuated [15], the equivalent deletion in the I/II chimeric backbone remained lethal (specific data not shown here). However, unlike pathological fever associated with severe clinical signs and mortality, the transient fever observed with JX23-02ΔD250R was self-limiting and followed by full recovery, indicating it represens an immunogenic activation reaction rather than pathogenic residual virulence.

Viremia is generally considered a significant indicator of immune function. Typically, Where high viremia often correlates with immune deficiency and low viremia with overactive immunity [25]. However, we observed a notable phenomenon:sustained low-level viremia in the JX23-02ΔD250R group during the immunization period was significantly correlated with superior viral clearance in tissues post-challenge. This aligns with the “memory inflation” theory described by Klenerman and Oxenius in viral models, where persistent low-level antigen stimulation maintains an expanded pool of functional effector memory T cells without inducing T-cell exhaustion (typically caused by high antigen loads) [26]. Therefore, we posit that the long-term low-level viremia induced by JX23-02ΔD250R represents moderate in vivo replication of the vaccine strain, rather than a safety deficit, which is crucial for triggering a robust systemic immune response. Conversely, the SY18ΔD250R group exhibited no detectable viremia, suggesting potential over-attenuation; this insufficient immunogenicity likely led to its failure against broader viral challenges.

The induction of effective protection relies intrinsically on the synergy between humoral and cellular immunity. While ASFV antibodies often lack neutralizing activity [27–29], they still play a beneficial role, and cell-mediated immunity – particularly the role of IFN-γ in activating immune cells – is paramount for viral clearance [31,32]. The discrepancy between the limited in vitro replication of SY18ΔD250R (suggesting a “multi-cycle replication defect” suppressed by rapid host antiviral states) and its 80% protective efficacy in vivo is noteworthy. Even this restricted replication was sufficient to induce protective immunity in most pigs. However, the sole non-survivor (Pig #60) in the SY18ΔD250R group remained consistently negative for p54 antibodies and exhibited minimal IFN-γ responses. This case underscores that robust antibody responses and adequate cellular immunity are both indispensable requirements for complete immune protection. Furthermore, it suggests that immunization failure driven by individual differences in immune reactivity (as seen in Pig #60) is a natural and widespread biological phenomenon.

Achieving cross-protection has consistently been the most significant challenge in ASFV vaccine development. While several live attenuated vaccines (LAVs) demonstrated varying degrees of cross-protection [30–32], their protective efficacy against the I/II recombinant strain remains unknown. Notably, although commercial LAVs like ASFV-G-ΔI177L and ASFV-G-ΔMGF are licensed in Vietnam, their practical application faces dual challenges. On one hand, vaccines like the ASFV-G-ΔMGF can persist and evolve in vaccinated pigs, leading to vaccine-derived recombinant strains that revert to virulence and cause severe clinical diseases (e.g. abortion and ulcerative dermatitis) in unvaccinated pigs [33]. On the other hand, current commercial vaccines fail to protect against newly emerging genotype I/II recombinant strains. In stark contrast, JX23-02ΔD250R demonstrates superior broad-spectrum efficacy. It successfully provided 100% cross-protection against both the parental Genotype I/II recombinant strain and prevailing Genotype II strains (SY18 and HuB20), characterized by undetectable or low-level viral shedding.

**Figure 8.**
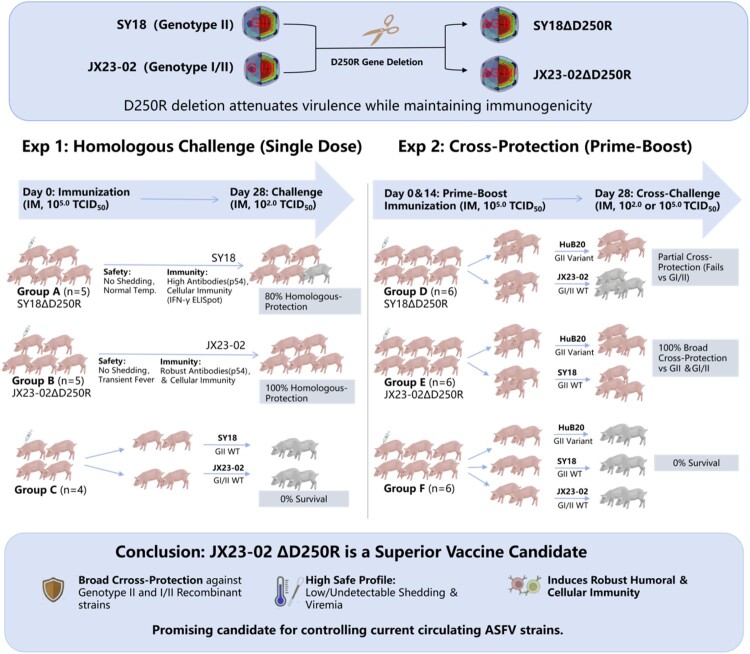
Schematic illustration of the development and comparative efficacy of *D250R*-deleted ASFV vaccine candidates.

In summary, by targeted the virulence-associated *D250R* gene, this study highlights JX23-02ΔD250R as a highly potent and broadly protective vaccine candidate capable of mitigating the complex epidemic caused by co-circulating ASFV genotypes (Figure 8). However, we also acknowledge the limitations of the current study that dictate our future research directions. Although JX23-02ΔD250R is rapidly cleared in vivo post-vaccination, evaluating its safety in pregnant sows at different gestational stages remains a top priority. Furthermore, to unequivocally ensure that the vaccine does not revert to virulence or persist in the environment, we will conduct rigorous long-term genetic stability tests and continuously monitor shedding risks, thereby guaranteeing its long-term safety under diverse and complex field conditions.

## Materials and methods

### Cells and viruses

Porcine alveolar macrophages (PAMs) and bone marrow-derived macrophages (BMDMs) were isolated from lung and bone tissues of 2–3 month-old healthy Landrace pigs, respectively. The method for PAMs isolation can be found in published research [15]. The BMDMs isolation procedure is briefly described as follows: The meat was removed, and the leg and rib bones were sectioned into 5 cm lengths. Using a syringe and PBS, cells were flushed from the bone marrow. The cell suspension was filtered through cell filter with a pore size of 100 μm (Falcon, USA), and cells were collected by centrifugation. Red blood cells were lysed one to two times using a red blood cell lysis buffer (TBD science, China). After centrifugation, the cell pellet was resuspended and washed 3–4 times with PBS containing 2% fetal bovine serum (FBS). Finally, cells were resuspended, counted, and cultured in medium supplemented with 10% FBS (Cao Yuan Lv Ye, China) and 10 μg/mL GM-CSF [34]. After differentiation, BMDMs were digested, cryopreserved, and stored for use.

The ASFV strain SY18 is a genotype II virulent strain (accession number: MH766894), isolated from the first reported ASF outbreak in China in 2018 [7]. The ASFV HuB20 strain (Accession No. MW521382), a Genotype II variant isolated in 2020 from Hubei Province, China, is characterized by a natural large-fragment deletion within the EP153R/EP402R genes compared to the SY18 strain. Consequently, this strain exhibits a non-hemadsorbing (HAD-negative) phenotype, attenuated virulence, and a delayed time to death of approximately 22 days post-infection (dpi). The SY18ΔD250R strain (Accession No.: PX911504) is a deletion mutant of SY18, lacking D250R. The ASFV strain JX23-02 (Accession No: PP712069) is a recombinant virulent strain of genotypes I and II [10], isolated in Jiangxi Province, China, in 2023. The JX23-02ΔD250R strain (Accession No.: PX911505) is a deletion mutant of JX23-02 derived from this strain, lacking *D250R*.

### Conservation Analysis of *D250R* Gene

ASFV genomes were downloaded from the NCBI website (https://www.ncbi.nlm.nih.gov/). The conservation of *D250R* amino acid residues across different ASFV genotypes and virulent strains was analyzed using the MAFFT website (https://mafft.cbrc.jp/alignment/server/) and Jalview 2.11.5.0 software.

### Construction of deletion mutants

Homologous arm plasmids were first constructed to facilitate the knockout of the ASFV *D250R* gene. Since the homologous arms for the SY18 and JX23-02 strains exhibited 100% homology at the *D250R* locus, a single homologous arm plasmid was used for gene knockout. Using the SY18 genomic DNA as a template, DNA fragments of 1.2 kb upstream (*D250R* LR) and 1.2 kb downstream (*D250R* RR) of the *D250R* gene were amplified by PCR. The *p72* promoter [35], mCherry gene sequence, and SV40 polyA terminator sequence were amplified from existing laboratory plasmids. The amplified sequences were then assembled in the order of *D250R* LR-*p72* Promoter-mCherry-SV40 polyA-*D250R* RR and cloned into the pMD18-T vector, yielding the recombinant plasmid designated T-Δ*D250R*-mCherry. The T-Δ*D250R*-mCherry plasmid was transfected into BMDMs using a golden transfection reagent (Golden Transfer Science and Technology, China). After 4 hours, the BMDMs were infected with ASFV SY18 and ASFV JX23-02 at a multiplicity of infection (MOI) of 3. At 24 hours post-infection (hpi), red fluorescent cells were observed under a fluorescence microscope. Purified red fluorescent cells were obtained by the endpoint dilution method, ultimately yielding the SY18ΔD250R and JX23-02ΔD250R strains. The complete purification of the deletion mutants was confirmed by PCR. The primers to confirm the purification of SY18ΔD250R and JX23-02ΔD250R are Identi-F: 5’-CATATGGATACTGCCATGCAGC-3’ and Identi-R: 5’-CATATTGGGTTCCTCCAACGACTTACATAACATTGC-3’.

### Genome sequencing

Genomic DNA was extracted from the SY18ΔD250R and JX23-02ΔD250R virus preparations using the Axygen Body Fluid Virus DNA/RNA Extraction Kit (Corning, USA). Next-generation sequencing (NGS) was performed by Novogene on the Illumina NovaSeq 6000 platform with a PE150 configuration to achieve full-length genome assembly of both *D250R* gene-deleted strains.

### In vitro growth characteristics analysis

The in vitro replication level of SY18ΔD250R and JX23-02ΔD250R were further compared with their respective wild-type viruses. PAMs were infected with SY18ΔD250R, JX23-02ΔD250R, and the wild type SY18, JX23-02 at an MOI of 0.01. Infected cell culture supernatants were collected at 0, 2, 6, 12, 24, 36, 48, 60, 72, 96, 120, and 144 hpi. Samples were subjected to three freeze–thaw cycles. The supernatant after centrifugation was serially diluted from 10^1.0^ to 10^7.0^. Each dilution (100μL/well) were added to PAMs in 96-well plates, with 8 replicates per dilution. At 96 dpi, the number of infected PAMs was determined by direct immunofluorescence staining. Viral titres at each time point were calculated using the Reed-Muench method. Based on the virus harvest time and TCID_50_, viral growth curves were plotted.

### Experiment 1: animal immunization and homologous challenge protection trial

**Figure 9.**
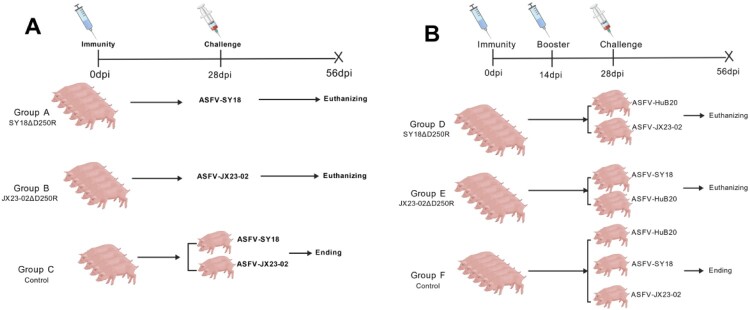
Experimental design for animal studies. A: Experiment 1, Animal Immunization and Homologous Challenge Protection Trial. B: Experiment 2, Animal Immunization and Heterologous Challenge Protection Trial.

To evaluate the virulence and protective efficacy of the *D250R* gene-deleted strain, infection and homologous challenge protection trials were conducted with SY18ΔD250R and JX23-02ΔD250R. 14 Large White pigs, weighing approximately 15 kg each, were randomly divided into three groups (A, B, and C). Group A (5 pigs, IDs #56, #57, #58, #59, #60) was intramuscularly inoculated with 10^5.0^ TCID_50_ of SY18ΔD250R. Group B (5 pigs, IDs #94, #95, #96, #97, #98) was intramuscularly inoculated with 10^5.0^ TCID_50_ of JX23-02ΔD250R. Group C (4 pigs, IDs #14, #15, #16, #17) was intramuscularly injected with 1 mL of physiological saline. These served as challenge control groups for the SY18 and JX23-02, respectively, with a challenge dose of 100 TCID_50_. The immunization observation period was 28 days. An experimental schematic is shown in Figure 9(A). Daily records of clinical signs and body temperature changes were maintained for the immunized pigs. Oral swabs, anal swabs, anticoagulated blood, and serum were collected periodically for virus load and antibody level detection. Following the challenge observation period, surviving pigs were euthanized, necropsied, and tissues including the heart, liver, spleen, and lymph nodes were collected for tissue virus load determination. Fixed tissues were processed for pathological analysis.

### Experiment 2: animal immunization and heterologous challenge protection trial

To assess the cross-protective efficacy of the *D250R* gene-deleted strain, 18 Large White pigs, weighing approximately 15 kg each, were randomly allocated into three groups (D, E, and F). Group D (6 pigs, IDs #61, #62, #63, #64, #65, #66) was intramuscularly inoculated with 10^5.0^ TCID_50_ of SY18ΔD250R, followed by a booster immunization with the same dose and route 14 days after the primary immunization. Group E (6 pigs, IDs #99, #100, #101, #102, #103, #104) was intramuscularly inoculated with 10^5.0^ TCID_50_ of JX23-02ΔD250R, followed by a booster immunization with the same dose and route 14 days after the primary immunization. Group F (6 pigs, IDs #18, #19, #20, #21, #22, #23) served as control pigs and were intramuscularly injected with 1 mL of physiological saline. After 28 days, pigs in Group D were challenged with 10^5.0^ TCID_50_ of HuB20 and 100 TCID_50_ of JX23-02. Pigs in Group E were challenged with 10^5.0^ TCID_50_ of HuB20 and 100 TCID_50_ of SY18. Pigs in Group F were challenged with 10^5.0^ TCID_50_ of HuB20, 100 TCID_50_ of SY18, and 100 TCID_50_ of JX23-02. An experimental schematic is shown in Figure 9(B). Daily records of clinical signs and body temperature changes were maintained during the immunization and challenge periods, and survival rates were calculated. Pigs #61, #62 and #63 in Group D were challenged with the HuB20 strain, and pigs #64, #65 and #66 were challenged with the JX23-02 strain. Pigs #99, #100 and #101 in Group E were challenged with the HuB20 strain, and pigs #102, #103 and #104 were challenged with the SY18 strain. Pigs #18 and #19 in Group G were challenged with the HuB20 strain, pigs #20 and #21 with the SY18 strain, and pigs #22 and #23 with the JX23-02 strain.

### Quantitative PCR detection

ASFV in pig tissues, blood, and swab samples was detected using a TaqMan probe-based method. Initially, ASFV DNA was extracted from tissues, blood, and swabs using a genomic DNA extraction kit (Vazyme, China). The specific primers and probe recommended by the OIE targeting the *B646L* (*p72*) gene were utilized: forward primer (OIE-*p72* F): 5’-CTGCTCATGGTATCAATCTTATCGA-3’; reverse primer (OIE-*p72* R): 5’-GATACCACAAGATCRGCCGT-3’; and TaqMan probe (OIE-*p72*-probe): 5’-FAM-CCACGGGAGGAATACCAACCCAGTG-TAM-3’. To quantify viral load, the pMD18T-*p72* standard plasmid (4751 bp in length; sequence provided in Supplementary File 1), which is maintained in our laboratory, was used to generate a standard curve. Following concentration measurement and conversion to copy number, ten-fold serial dilutions ranging from 10^1.0^-10^8.0^ copies/μL were prepared. The resulting regression equation was y = −3.43x + 40.17. Where y represents the cycle threshold (Ct) value and x represents the common logarithm of the viral copy number (log_10_ copies). Viral copy numbers were calculated by substituting the Ct values obtained from qPCR into the aforementioned equation.

### ASFV-*p54* antibody detection

For Experiment 1 pigs, blood was collected on days 0, 3, 7, 14, 21, and 28 post-immunization, and on days 3, 7, 14, 21, and 28 post-challenge. Serum was separated for ASFV *p54* antibody detection, following the methodology described in published research.

### Pathological analysis

Heart, liver, spleen, lungs, kidneys, thymus, and lymph nodes were fixed in 4% paraformaldehyde. Tissue sectioning, staining, and image scanning were outsourced to Prattzer Laboratory. The process involved dehydration, paraffin embedding, sectioning, and baking of the fixed tissues. Tissues were deparaffinized with xylene, rehydrated sequentially with ethanol (100%-75%) and distilled water, stained with hematoxylin, washed, and then stained with eosin. Differentiation and dehydration were performed with ethanol, followed by clearing in xylene. After coverslipping, tissues were observed under a microscope and scanned to generate images for pathological analysis.

### Enzyme-Linked immunospot (ELISpot) assay

For Experiment 1 pigs, anticoagulated blood was collected on days 7, 14, 21, and 28 post-immunization (dpi). Peripheral blood mononuclear cells (PBMCs) were isolated using a peripheral blood mononuclear cell isolation kit (Solarbio, China). The cell concentration was adjusted to 2×10^6.0^ cells/mL. The detailed detection procedure was performed according to the kit's instructions(Cellular Technology Limited, USA). Briefly, inactivated ASFV SY18 and Concanavalin A (ConA) were added to a culture plate. PBMCs were added at 100 μL/well and incubated statically for 20 hours. The culture plate was washed with PBST. Detection solution for porcine *IFN-γ* was added and incubated at room temperature for 2 hours. The plate was washed with PBST, followed by the addition of Strep-AP diluent and incubation at room temperature for 0.5 hours. Colour development was achieved using a blue substrate solution for 15 minutes, followed by rinsing with tap water. After air-drying, the number of blue spots was counted.

### Statistical analysis

Statistical significance was determined by the Holm-Sidak test, with P values < 0.05 considered statistically significant. Similar results were obtained in at least three independent experiments. Multiple comparisons were performed using the Two-way ANOVA method with GraphPad Prism 8.0 software to analyze for statistically significant differences between groups.

### Compliance and ethics

The study was conducted according to standard procedures approved by The Animal Welfare and Ethics Committee of Changchun Veterinary Research Institute and the animal bio-safety level 3 (ABSL-3) lab (review ID: IACUC of AMMS-11-2024-058, approved at August 30, 2024). All opinions expressed in this paper are those of the authors and do not necessarily reflect the policies and views of the Ministry of Agriculture and Rural Affairs of China. RLH, YYZ, TC, et al have a patent application filed by Changchun Veterinary Research Institute, Chinese Academy of Agricultural Sciences for ASFV SY18ΔD250R and JX23-02ΔD250R as a live attenuated vaccine for African swine fever.

## Supplementary Material

Supplementary File .docx

Supplementary_File_clean.docx
