# Supplementary material for: Hybrid genotype I and II ASFV D250R deletions confer protection against parental and genotype II strains and elicit potent immune response
Source: Emerg Microbes Infect. 2026 Mar 13;15(1):2640697. doi: 10.1080/22221751.2026.2640697 (PMC12990284; doi:10.1080/22221751.2026.2640697)
Supplement: Supplementary File .docx [file TEMI_A_2640697_SM9453.docx]

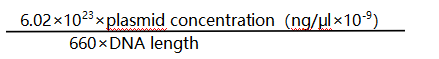
Specifically, we utilized the previously constructed pMD18T-*p72* recombinant plasmid from our laboratory as the standard plasmid. The full-length *p72* gene was obtained via PCR, and the purified *p72* gene fragment was cloned into the pMD18-T vector to generate the recombinant plasmid pMD18T-*p72* (4,751 bp in total length). The plasmid was extracted using a plasmid extraction kit, and its concentration was measured. The plasmid copy number was calculated using the formula:

copies/μL=

Based on the known copy number, the plasmid was serially diluted ten-fold to obtain plasmid dilutions ranging from 10¹ to 10⁸ copies/μL for constructing the standard curve. The resulting regression equation was y = -3.43x + 40.17, where y represents the cycle threshold (Ct) value, and x represents the common logarithm (log₁₀) of the viral copy number. The viral copy number was calculated by substituting the Ct values obtained from qPCR into the above equation.

The standard plasmid pMD18T-p72 is 4751 bp in length：

TCGCGCGTTTCGGTGATGACGGTGAAAACCTCTGACACATGCAGCTCCCGGAGACGGTCACAGCTTGTCTGTAAGCGGATGCCGGGAGCAGACAAGCCCGTCAGGGCGCGTCAGCGGGTGTTGGCGGGTGTCGGGGCTGGCTTAACTATGCGGCATCAGAGCAGATTGTACTGAGAGTGCACCATATGCGGTGTGAAATACCGCACAGATGCGTAAGGAGAAAATACCGCATCAGGCGCCATTCGCCATTCAGGCTGCGCAACTGTTGGGAAGGGCGATCGGTGCGGGCCTCTTCGCTATTACGCCAGCTGGCGAAAGGGGGATGTGCTGCAAGGCGATTAAGTTGGGTAACGCCAGGGTTTTCCCAGTCACGACGTTGTAAAACGACGGCCAGTGCCAAGCTTGCATGCCTGCAGGTCGACGATCAATAATTTTAAGCTGATCGTTAATTAATTTTTGGTTTAACTCTTTGTTATTATCAAGATCCTTCGCATAAACCGCCATATTTAATAAAAACAATAAATTATTTTTATAACATTATATATGGCATCAGGAGGAGCTTTTTGTCTTATTGCTAACGATGGGAAGGCCGACAAGATTATATTGGCCCAAGACTTGCTGAATAGCAGGATCTCTAACATTAAAAATGTGAACAAAAGTTATGGGAAACCCGATCCCGAACCCACTTTGAGTCAAATCGAAGAAACACATTTGGTGCATTTTAATGCGCATTTTAAGCCTTATGTTCCAGTAGGGTTTGAATACAATAAAGTACGCCCGCATACGGGTACCCCCACCTTGGGAAACAAGCTTACCTTTGGTATTCCCCAGTACGGAGACTTTTTCCATGATATGGTGGGCCATCATATATTGGGTGCATGTCATTCATCCTGGCAGGATGCTCCGATTCAGGGCACGTCCCAGATGGGGGCCCATGGGCAGCTTCAAACGTTTCCTCGCAACGGATATGACTGGGACAACCAAACACCCTTAGAGGGCGCCGTTTACACGCTTGTAGATCCTTTTGGAAGACCCATTGTACCCGGCACAAAGAATGCGTACCGAAACTTGGTTTACTACTGCGAATACCCCGGAGAACGACTTTATGAAAACGTAAGATTCGATGTAAATGGAAATTCCCTAGACGAATATAGTTCGGATGTCACAACGCTTGTGCGCAAATTTTGCATCCCAGGGGATAAAATGACTGGATATAAGCACTTGGTTGGCCAGGAGGTATCGGTGGAGGGAACCAGTGGCCCTCTCCTATGCAACATTCATGATTTGCACAAGCCGCACCAAAGCAAACCTATTCTTACCGATGAAAATGATACGCAGCGAACGTGTAGCCATACCAACCCGAAATTTCTTTCACAGCATTTTCCCGAGAACTCTCACAATATCCAAACAGCAGGTAAACAAGATATTACTCCTATCACGGACGCAACGTATCTGGACATAAGACGTAATGTTCATTACAGCTGTAATGGACCTCAAACCCCTAAATACTATCAGCCCCCTCTTGCGCTCTGGATTAAGTTGCGCTTTTGGTTTAATGAGAACGTGAACCTTGCTATTCCCTCAGTATCCATTCCCTTCGGCGAGCGCTTTATCACCATAAAGCTTGCATCGCAAAAGGATTTGGTGAATGAATTTCCTGGACTTTTTGTACGCCAGTCACGTTTTATAGCTGGACGCCCCAGTAGACGCAATATACGCTTTAAACCATGGTTTATCCCAGGAGTCATTAATGAAATCTCGCTCACGAATAATGAACTTTACATCAATAACCTGTTTGTAACCCCTGAAATACACAACCTTTTTGTAAAACGCGTTCGCTTTTCGCTGATACGTGTCCATAAAACGCAGGTGACCCACACCAACAATAACCACCACGATGAAAAACTAATGTCTGCTCTTAAATGGCCCATTGAATATATGTTTATAGGATTAAAACCTACCTGGAACATCTCCGATCAAAATCCTCATCAACACCGAGATTGGCACAAGTTCGGACATGTTGTTAACGCCATTATGCAGCCCACTCACCACGCAGAGATAAGCTTTCAGGATAGAGATACAGCTCTTCCAGACGCATGTTCATCTATATCTGATATTAGCCCCGTTACGTATCCGATCACATTACCTATTATTAAAAACATTTCCGTAACTGCTCATGGTATCAATCTTATCGATAAATTTCCATCAAAGTTCTGCAGCTCTTACATACCCTTCCACTACGGAGGCAATGCGATTAAAACCCCCGATGATCCGGGTGCGATGATGATTACCTTTGCTTTGAAGCCACGGGAGGAATACCAACCCAGTGGTCATATTAACGTATCCAGAGCAAGAGAATTTTATATTAGTTGGGACACGGATTACGTGGGGTCTATCACTACGGCTGATCTTGTGGTATCGGCATCTGCTATTAACTTTCTTCTTCTTCAGAACGGTTCAGCTGTGCTGCGTTACAGTACCTAAATCTCTAGAGGATCCCCGGGTACCGAGCTCGAATTCGTAATCATGGTCATAGCTGTTTCCTGTGTGAAATTGTTATCCGCTCACAATTCCACACAACATACGAGCCGGAAGCATAAAGTGTAAAGCCTGGGGTGCCTAATGAGTGAGCTAACTCACATTAATTGCGTTGCGCTCACTGCCCGCTTTCCAGTCGGGAAACCTGTCGTGCCAGCTGCATTAATGAATCGGCCAACGCGCGGGGAGAGGCGGTTTGCGTATTGGGCGCTCTTCCGCTTCCTCGCTCACTGACTCGCTGCGCTCGGTCGTTCGGCTGCGGCGAGCGGTATCAGCTCACTCAAAGGCGGTAATACGGTTATCCACAGAATCAGGGGATAACGCAGGAAAGAACATGTGAGCAAAAGGCCAGCAAAAGGCCAGGAACCGTAAAAAGGCCGCGTTGCTGGCGTTTTTCCATAGGCTCCGCCCCCCTGACGAGCATCACAAAAATCGACGCTCAAGTCAGAGGTGGCGAAACCCGACAGGACTATAAAGATACCAGGCGTTTCCCCCTGGAAGCTCCCTCGTGCGCTCTCCTGTTCCGACCCTGCCGCTTACCGGATACCTGTCCGCCTTTCTCCCTTCGGGAAGCGTGGCGCTTTCTCATAGCTCACGCTGTAGGTATCTCAGTTCGGTGTAGGTCGTTCGCTCCAAGCTGGGCTGTGTGCACGAACCCCCCGTTCAGCCCGACCGCTGCGCCTTATCCGGTAACTATCGTCTTGAGTCCAACCCGGTAAGACACGACTTATCGCCACTGGCAGCAGCCACTGGTAACAGGATTAGCAGAGCGAGGTATGTAGGCGGTGCTACAGAGTTCTTGAAGTGGTGGCCTAACTACGGCTACACTAGAAGAACAGTATTTGGTATCTGCGCTCTGCTGAAGCCAGTTACCTTCGGAAAAAGAGTTGGTAGCTCTTGATCCGGCAAACAAACCACCGCTGGTAGCGGTGGTTTTTTTGTTTGCAAGCAGCAGATTACGCGCAGAAAAAAAGGATCTCAAGAAGATCCTTTGATCTTTTCTACGGGGTCTGACGCTCAGTGGAACGAAAACTCACGTTAAGGGATTTTGGTCATGAGATTATCAAAAAGGATCTTCACCTAGATCCTTTTAAATTAAAAATGAAGTTTTAAATCAATCTAAAGTATATATGAGTAAACTTGGTCTGACAGTTACCAATGCTTAATCAGTGAGGCACCTATCTCAGCGATCTGTCTATTTCGTTCATCCATAGTTGCCTGACTCCCCGTCGTGTAGATAACTACGATACGGGAGGGCTTACCATCTGGCCCCAGTGCTGCAATGATACCGCGAGACCCACGCTCACCGGCTCCAGATTTATCAGCAATAAACCAGCCAGCCGGAAGGGCCGAGCGCAGAAGTGGTCCTGCAACTTTATCCGCCTCCATCCAGTCTATTAATTGTTGCCGGGAAGCTAGAGTAAGTAGTTCGCCAGTTAATAGTTTGCGCAACGTTGTTGCCATTGCTACAGGCATCGTGGTGTCACGCTCGTCGTTTGGTATGGCTTCATTCAGCTCCGGTTCCCAACGATCAAGGCGAGTTACATGATCCCCCATGTTGTGCAAAAAAGCGGTTAGCTCCTTCGGTCCTCCGATCGTTGTCAGAAGTAAGTTGGCCGCAGTGTTATCACTCATGGTTATGGCAGCACTGCATAATTCTCTTACTGTCATGCCATCCGTAAGATGCTTTTCTGTGACTGGTGAGTACTCAACCAAGTCATTCTGAGAATAGTGTATGCGGCGACCGAGTTGCTCTTGCCCGGCGTCAATACGGGATAATACCGCGCCACATAGCAGAACTTTAAAAGTGCTCATCATTGGAAAACGTTCTTCGGGGCGAAAACTCTCAAGGATCTTACCGCTGTTGAGATCCAGTTCGATGTAACCCACTCGTGCACCCAACTGATCTTCAGCATCTTTTACTTTCACCAGCGTTTCTGGGTGAGCAAAAACAGGAAGGCAAAATGCCGCAAAAAAGGGAATAAGGGCGACACGGAAATGTTGAATACTCATACTCTTCCTTTTTCAATATTATTGAAGCATTTATCAGGGTTATTGTCTCATGAGCGGATACATATTTGAATGTATTTAGAAAAATAAACAAATAGGGGTTCCGCGCACATTTCCCCGAAAAGTGCCACCTGACGTCTAAGAAACCATTATTATCATGACATTAACCTATAAAAATAGGCGTATCACGAGGCCCTTTCGTC
